# Why is nanomedicine needed to treat disorders of the endometrium?

**DOI:** 10.1002/ctm2.70811

**Published:** 2026-09-11

**Authors:** Saed Abbasi, Laura M. Ensign

**Affiliations:** ^1^ Centre for Nanomedicine at the Wilmer Eye Institute The Johns Hopkins University School of Medicine Baltimore Maryland USA

1

The endometrium, the inner lining of the uterus, is important as it supports embryo implantation. Dysfunction of the endometrium can lead to embryo implantation failure and pregnancy loss. Thin endometrium (TE) is a condition characterised by the endometrial thickness measuring below 7 mm during the mid‐luteal phase of the menstrual cycle.^1^ TE can be spontaneous or caused by gynaecologic conditions, including uterine adhesions, fibroids, infections, and Asherman's Syndrome.^2^ Current treatments for TE include hormonal or vasodilator therapy, but none is Food and Drug Administration‐approved.^2^

While the underlying mechanisms behind TE are multifactorial, newer treatment options that are aimed at regenerating the endometrium before embryo transfer during assisted reproductive therapy (ART) have shown promise in preclinical and clinical studies. Of these methods, systemic or intrauterine administration of growth factors or cytokines has been used to increase endometrial thickness, repair endometrial injury, and improve embryo implantation rates.^3^, ^4^ However, recombinant protein therapy comes with several challenges, including the difficulty of manufacturing and purification, lack of innate protein posttranslational modifications, and poor tissue penetration and targeting.^5^

Over the past few years, the use of messenger RNA (mRNA) has revolutionized the fields of gene editing and vaccine development, as mRNA offers tremendous advantages over recombinant protein therapy, mainly for two key reasons: (i) proteins synthesized within target cells undergo authentic post‐translational modifications and extracellular secretion patterns, which cannot be replicated by recombinant proteins produced in heterologous expression systems^6^ (ii) mRNA enables transient protein expression, peaking within hours and persisting for hours to days^7^. Unlike gene augmentation therapy using viral vectors, mRNA transfection is more transient, enabling repeated administration while allowing the effects to be reversed upon discontinuation. Taken together, mRNA‐based protein expression could be ideal to recapitulate the physiological, transient production of hormones and cytokines in the endometrium.

Although mRNA has strong therapeutic potential, its in vivo application is hindered by several barriers. First, mRNA is highly susceptible to degradation by nucleases present in biological fluids.^8^ Second, mRNA is highly negatively charged because of its phosphate backbone and has a molecular weight of hundreds of thousands of daltons, making it difficult to cross cell membranes and enter cells on its own. Therefore, protecting mRNA from degradation and facilitating its intracellular delivery are essential for enabling it to exert its therapeutic effects.^9^

Nanomedicine is the application of nanotechnology to provide solutions in healthcare. Nanoparticles can be used to condense large mRNA molecules into their cores, protect the mRNA from degradation, boost its intracellular uptake, and facilitate its endosomal escape. While there has been a myriad of materials developed for making mRNA nanoparticles, delivery systems based on lipid nanoparticles (LNPs) have proven both safety and efficacy in humans and are compatible with a wide range of applications such as infectious disease and cancer vaccination, gene editing, and therapeutic protein supplementation.^10^ LNPs can also be used to guide their payloads to specific target tissues and cells.^11^ For example, attaching a targeting moiety on the surface of the LNP can facilitate its homing to certain receptors that are overexpressed on cancer cells, thereby improving drug concentrations within the tumor tissue and reducing its off‐target side effects.^12^

We recently developed the first targeted mRNA‐LNP to the endometrium.^13^ To do so, we leveraged the overexpressed integrin receptors on the endometrium during the window of implantation (WOI) to improve mRNA uptake by endometrial cells. We conjugated our LNPs with arginylglycylaspartic acid (RGD) to enhance mRNA‐LNP uptake by the integrin pathway. Using mice at day 5 post coitus (day 5 p.c.) to model the WOI in humans, we delivered RGD‐conjugated and unconjugated mRNA‐LNPs by intrauterine infusions and evaluated protein expression efficiency in the uterus and other organs, including the liver, spleen, and cervix (Figure 1). We found that the LNP modified with 5 mol% RGD‐Lipid (LNP B) increased luciferase expression in the uterus by 4‐fold compared to unconjugated LNP (LNP A) (Figure 1(A)). However, infusing the unconjugated or RGD‐conjugated mRNA‐LNPs (LNPs A or B) in unmated mice (outside the WOI) resulted in indistinguishable luciferase expression levels in the uterus (Figure 1(B)). This emphasizes the role of RGD‐conjugation in enhancing mRNA‐LNP delivery to the endometrium when infused during the WOI, where integrin receptors are overexpressed. Interestingly, we also observed that LNP B reduced luciferase expression in the liver and spleen compared to LNP A by up to a hundred‐fold, whether it was infused during or outside the WOI (Figure 1(A,B)). This highlights the unexpected role of the RGD‐lipid in not only enhancing mRNA delivery to the endometrium when infused during the WOI but also reducing liver and spleen tropism in treated animals.^13^

**FIGURE 1:**
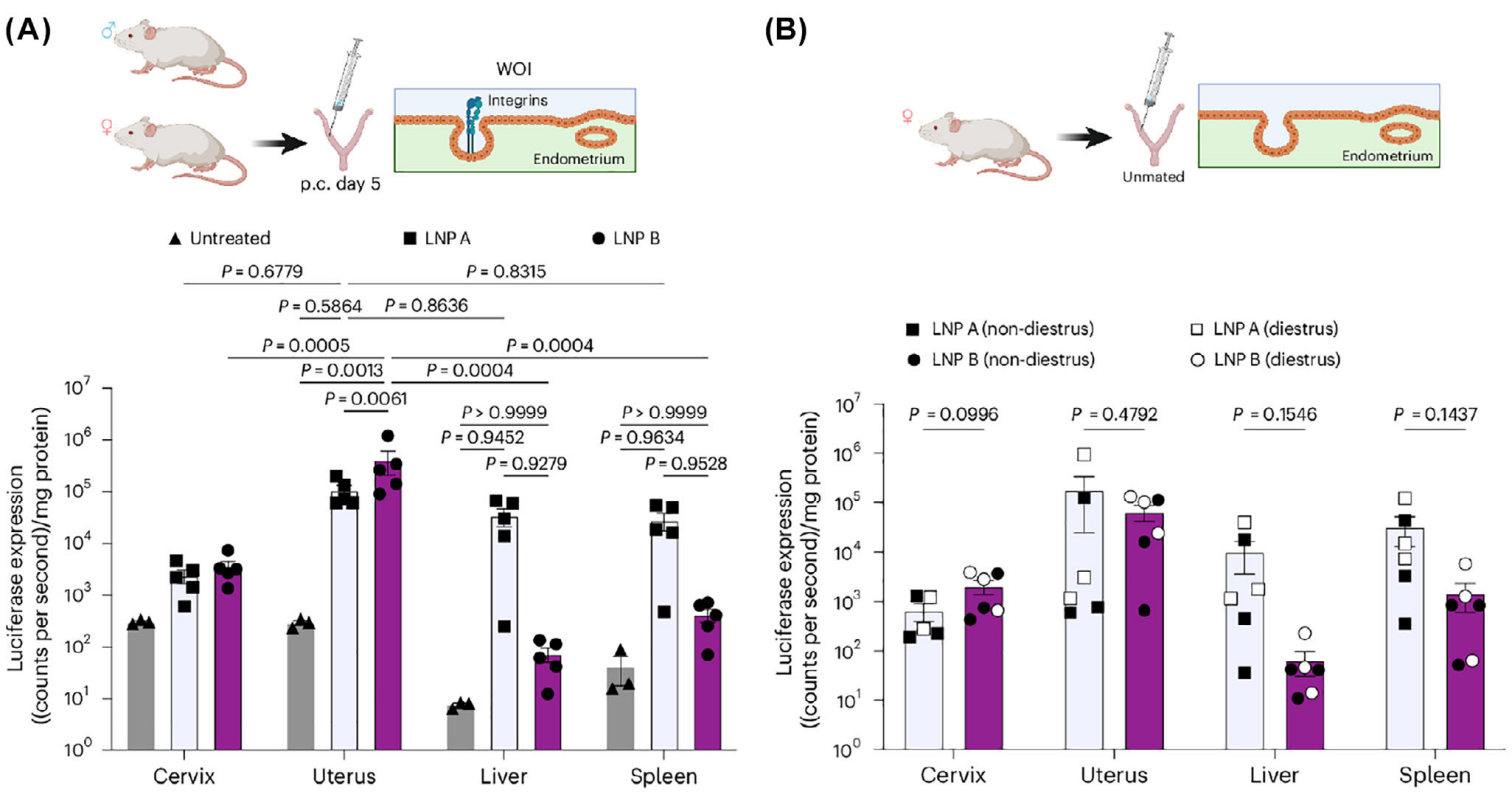
The in vivo delivery efficiency of arginylglycylaspartic acid (RGD)‐modified lipid nanoparticles (LNPs). (A) Luciferase expression normalised to mg protein in tissue homogenates 4 h post intrauterine infusion of 2 µg firefly luciferase messenger RNA (mRNA) loaded into LNPs in CD‐1 mice at p.c. day 5. LNP A contains 0 mol% RGD‐lipid, while LNP B contains 5 mol%. Treatments were infused into the mid‐distal right uterine horn. Data represent the mean ± s.e.m. (*n* = 5 animals for LNP groups and *n* = 3 for the untreated group). Two‐way analysis of variance (ANOVA) followed by Tukey's post hoc test. (B) Luciferase expression normalised to mg protein 4 h post intrauterine infusion of 2 µg fLuc mRNA loaded into LNPs in unmated CD‐1 mice (that is, outside the window of implantation [WOI]). LNP A contains 0 mol% RGD‐lipid, while LNP B contains 5 mol%. Mice in diestrus are represented by open circles or squares, whereas mice in non‐diestrus (oestrus, proestrus or metestrus) are represented by closed circles or squares. Data represent the mean ± s.e.m. (*n* = 6 mice). Two‐tailed, unpaired t‐tests. A statistically significant *p*‐value is below 0.05. Adapted.^13^

After demonstrating the improved targeting efficiency of LNP B to the endometrium, we used mRNA encoding granulocyte‐macrophage colony‐stimulating factor (GM‐CSF) as a therapeutic cytokine with endometrium‐regenerative function.^3^ First, we induced endometrial injury (TE) in the right uterine horn of each mouse by infusing ethanol, while the left uterine horn was uninjured and used as a control for successful embryo implantation after mating (Figure 2(A)). We found that the LNP B containing GM‐CSF mRNA was effective in restoring embryo implantation rates to levels comparable to the uninjured uterine horns (Figure 2(A)). We also found that the intrauterine infusion of LNP B containing GM‐CSF mRNA produced a much lower plasma exposure of the encoded protein compared to the intrauterine infusion of recombinant GM‐CSF (Figure 2(B)), further emphasising the advantage of the mRNA‐LNP technology in localising drug treatment.

**FIGURE 2:**
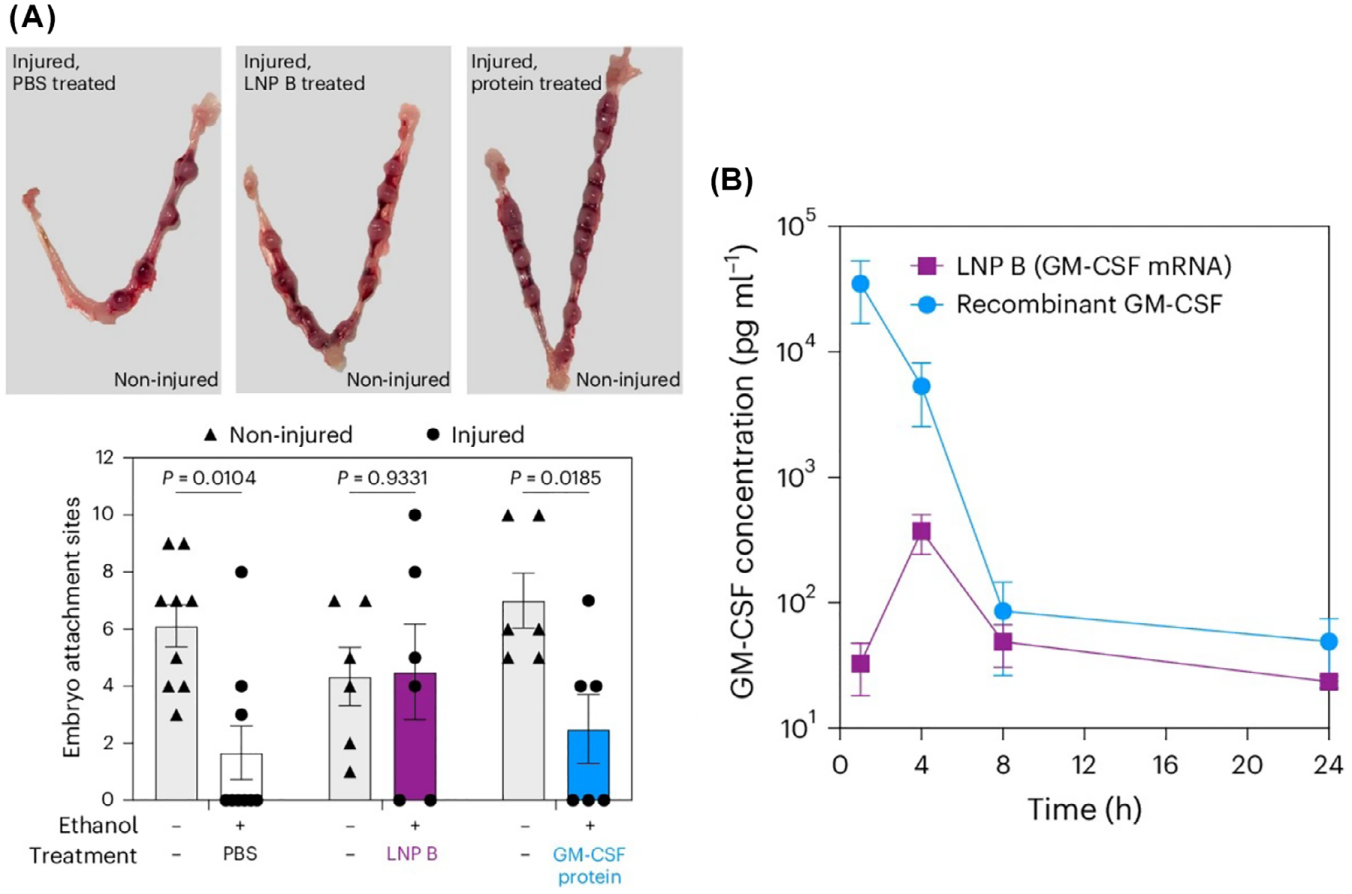
Targeted messenger RNA (mRNA)‐lipid nanoparticle (LNP) delivery in the endometrium restores fertility rates in mice. (A) LNP B (5 mol% arginylglycylaspartic acid [RGD]‐lipid) mediated the delivery of granulocyte‐macrophage colony‐stimulating factor (GM‐CSF) mRNA to the endometrium in a murine model of thin endometrium and restored embryo implantation rates. Data represent the mean ± s.e.m (n=6‐9 mice). Two‐tailed, paired t‐tests. A statistically significant *p*‐value is below 0.05. (B) GM‐CSF protein levels in the blood plasma following treatment with 4 µg GM‐CSF mRNA in LNP B or 3.6 µg mouse recombinant GM‐CSF in PBS infused into the right uterine horn on p.c. day 5. Data represent the mean ± s.e.m (n=3 animals). Adapted.^13^

Collectively, we demonstrated for the first time the utility of mRNA‐LNP for the treatment of TE. While GM‐CSF was used here as a model cytokine for proof‐of‐concept, mRNA can be readily engineered to encode virtually any therapeutic protein simply by changing its nucleotide sequence. Further, this platform delivery system can be used in the future for the potential treatment of a diverse range of endometrial dysfunctions, including endometrial cancers, endometriosis, and uterine fibroids. While the therapeutic utility in our study has been demonstrated in mice, rodents can poorly recapitulate the mechanisms of endometrial regeneration in humans, as rodents do not commonly menstruate.^14^ Therefore, testing in human‐derived endometrial cells and organoids represents the next step for ensuring the translational potential of our novel therapeutic strategy.

## CONFLICT OF INTEREST STATEMENT

Saed Abbasi and Laura M. Ensign are part of a patent application (PCT/US2025/043687) related to this work, which was filed by Johns Hopkins University.
